# A common variant in PIK3CG gene associated with the prognosis of heart failure

**DOI:** 10.1111/jcmm.70069

**Published:** 2024-09-08

**Authors:** Dong Hu, Lei Xiao, Shiyang Li

**Affiliations:** ^1^ Division of Cardiology, The Central Hospital of Wuhan, Tongji Medical College Huazhong University of Science and Technology Wuhan China; ^2^ Key Laboratory for Molecular Diagnosis of Hubei Province, The Central Hospital of Wuhan, Tongji Medical College Huazhong University of Science and Technology Wuhan China; ^3^ Division of Cardiology, Department of Internal Medicine, Tongji Hospital, Tongji Medical College Huazhong University of Science and Technology Wuhan China; ^4^ Department of Geriatrics Panzhihua Central Hospital Panzhihua China

**Keywords:** heart failure, PIK3CG, prognosis, variant

## Abstract

Phosphoinositide 3‐kinase γ (PI3Kγ) is G‐protein‐coupled receptor‐activated lipid kinase with both kinase‐dependent and kinase‐independent activity. Plenty of evidence have demonstrated that PI3Kγ participated in TAC and I/R‐induced myocardial remodelling and heart failure (HF). In this study, we tested the hypothesis that common variants in the PI3Kγ gene (*PIK3CG*) were associated with the prognosis of HF in the Chinese Han population. Through re‐sequencing and genotyping, we finally identified a common variant in the 3′UTR of *PIK3CG* strongly associated with the prognosis of HF in two‐stage population: adjusted *p* = 0.007, hazard ratio = 0.56 (0.36–0.85) in the first cohort and adjusted *p* = 0.024, hazard ratio = 0.39 (0.17–0.88) in the replicated cohort. A series of functional assays revealed that rs10215499‐A allele suppressed PIK3CG translation by facilitating has‐miR‐133a‐3p binding, but not the G allele. Subjects carrying the GG genotype showed higher mRNA and protein level than those with AA and AG genotype. Furthermore, overexpression of PIK3CG could protect AC16 from hypoxia/reoxygenation (H/R)‐induced apoptosis, while the case was opposite for PIK3CG silencing. In conclusion, common variant rs10215499 in the 3′‐UTR of *PIK3CG* might affect the prognosis of HF by interfering with miR‐133a‐3p binding and *PIK3CG* is a promising target for HF treatment in the future.

## 1 INTRODUCTION

Chronic heart failure (HF) is a complex clinical syndrome with multifactorial aetiology, presenting a significant global health challenge.^1^, ^2^ Despite considerable advancements in HF management over recent decades, mortality and rehospitalisation rates among HF patients remain alarmingly high.^3^, ^4^ With the aging of the population and various risk factors, the situation has been unoptimistic.^5^, ^6^ Better understanding of the genetic mechanism and finding novel therapeutic target for HF have emerged as a promising direction.^7^, ^8^

Phosphoinositide 3‐kinases (PI3Ks) are a family of enzymes with both protein and lipid kinase activity.^9^ The 3′‐position of phosphatidylinositol (PtdIns) (4,5) P2 could be phosphorylated by active PI3K and converted into phosphatidylinositol (3,4,5)‐trisphosphate (PIP3),^10^ followed by activation of a series of downstream targets including Akt/protein kinase B (PKB).^11^ According to the structure, function and substrate specificity, PI3Ks are divided into three different classes.^12^ A total of four different class I PI3Ks have been discovered, among which PI3Kα, PI3Kβ and PI3Kδ (class I_A_) isoenzymes are activated by receptor tyrosine kinase pathway.^9^ As a single member of Class I_B_, PI3Kγ heterodimerizes with a regulatory p87 or p101 subunit and is activated by βγ subunit of G‐proteins and functions downstream of G protein–coupled receptors (GPCRs).^13^, ^14^

PI3Kγ, which is primarily expressed in leukocytes and cardiovascular tissue,^9^, ^15^ has been reported to regulate a wide range of cellular process including cell growth, inflammation, motility and survival.^16^, ^17^, ^18^ Substantial evidence has demonstrated that PI3Kγ is involved in the internalization of activated β‐adrenergic receptor via its kinase activity.^19^, ^20^ Additionally, PI3Kγ acts as a scaffold protein to negatively regulate cAMP levels.^21^, ^22^, ^23^ Importantly, both the protein level and lipid kinase activity of PI3Kγ have been shown to be significantly upregulated in heart failure,^18^, ^19^, ^24^ suggesting a vital role of PI3Kγ in the pathological process of HF. Indeed, several reports have demonstrated that the PI3Kγ‐selective inhibitor AS605240 could ameliorate cardiac remodelling and dysfunction in TAC‐ and MI‐induced mouse HF models,^21^, ^25^ which indicated the deleterious role of kinase‐dependent function in myocardium. However, a study by Mauro et al. found that AS605240 significantly worsened MI‐induced cardiac dysfunction compared to control.^9^ On the contrary, the kinase‐independent scaffolding function of PI3Kγ was doubtlessly cardio‐protective as knock‐in for a catalytically inactive PI3Kγ (PI3Kγ‐KD) renders animals resistant to cardiac dysfunction under pathological conditions.^10^, ^19^, ^21^, ^22^ Overall, PI3Kγ consistently displayed favourable role in various HF models.^10^, ^11^, ^22^

Up to now, numerous genetic studies have examined the correlation between variants in the PI3Kγ gene *PIK3CG* and various disease states, including autistic disorder,^26^ subclinical atherosclerosis traits,^27^ platelet aggregation,^28^ poor responsiveness to clopidogrel,^29^ and others. Considering the importance of PI3Kγ in cardiac pathophysiology and the lack of relevant genetic investigations in HF, we hypothesize that *PIK3CG* polymorphism may serve as a potential prognostic indicator for HF.

## 2 METHODS

### 2.1 Study population

In the first cohort, a total of 2267 HF patients were successfully recruited for this study between 1 January 2009 and 31 October 2014 in Cardiology.

Division of Tongji Hospital in Wuhan. In the replicated cohort, 838 patients with chronic HF were used to validate the results observed in the first cohort. We defined cardiovascular deaths or cardiac transplantation as the primary end point. Details regarding the diagnostic and exclusion criteria for HF, data collection, and definition of risk factors have been previously described.^4^ The clinical characteristics of the study populations were summarized in Table 1.

**TABLE 1:** Baseline characteristics of the study population.

Characteristics	Two‐stage population	
	First cohort (*n* = 2267)	Replicated cohort (*n* = 838)
Male, %	1480 (65.3)	546 (65.2)
Age, years	59.7 ± 14.1	59.5 ± 14.3
Ischemic etiology^a^	1016 (44.8)	380 (45.3)
Glucose, mmol/L	6.6 ± 3.4	6.8 ± 3.4
TG, mmol/L	1.4 ± 1.2	1.4 ± 0.9
TC, mmol/L	4.0 ± 1.9	3.9 ± 1.3
HDL‐C, mmol/L	1.0 ± 0.6	1.0 ± 0.4
LDL‐C, mmol/L	2.4 ± 0.9	2.4 ± 0.9
Ejection fraction, %	46.4 ± 16.0	46.2 ± 16.5
Hypertension, %	1801 (79.4)	665 (79.4)
Diabetes, %	683 (30.1)	253 (30.2)
Hyperlipidemia, %	461 (20.3)	165 (19.7)
Smokers, %	848 (37.4)	297 (35.4)
*β*‐blocker use^a^	1818 (80.2)	667 (79.6)

*Note*: Data are expressed as means ± SD or percentages.

Abbreviations: HDL‐C, high‐density lipoprotein cholesterol; LDL‐C, low‐density lipoprotein cholesterol;TC, total cholesterol; TG, triglyceride.

^a^ Listed as number (%).

All protocols and methods were approved by the ethics committees of Tongji Hospital and conducted in accordance with the Declaration of Helsinki. We obtained written informed consents from all participants.

### 2.2 Genetic variation screening

*Genomic DNA was* extracted from peripheral venous blood samples obtained from 48 control participants, as previously reported.^30^ Sanger sequencing was performed to identify common variants in the PIK3CG gene. The promoter region and exons region in PIK3CG were amplified using polymerase‐chain‐reaction (PCR) and subsequently subjected to fluorescent dye‐terminator cycle sequencing. The primer sequences used for amplification can be found in Table S1.

### 2.3 Genotyping

Probe and primer sequences were designed using ABI Primer Expression 3.0 software and synthesized by Shanghai GeneCore Bio Technologies Co., Ltd, China. Genomic DNA was extracted from peripheral leucocytes as previously reported.^4^ The selected variants in *PIK3CG* gene were genotyped using the TaqMan assay on the TaqMan 7900HT Sequence Detection System (Applied Biosystems, Foster City, CA). Detailed procedures and conditions have been described previously.^31^ The probe and primer sequences are listed in Table S2.

### 2.4 Plasmids construction

We constructed the *PIK3CG* expression vectors using pcDNA3.1(+). The rs1129293 single nucleotide polymorphism (SNP) was introduced into this expression vector using Fast Mutagenesis System (Beijing TransGen Biotech Co., Ltd.) according to the manufacturer's instructions. Additionally, approximately 400 nucleotides located around rs3173908, rs10216210, rs10215499 and rs6150272 were respectively cloned into pMIR‐Report Luciferase. Detailed primer sequences were listed in Table S3.

### 2.5 Cell culture, transient transfection and luciferase activity assays

Human Embryonic Kidney 293 T cells (HEK293T) and AC16 were obtained from the American Type Culture Collection (ATCC) and cultured in Dulbecco's modified Eagle's medium supplemented with 10% fetal bovine serum (FBS; Gibco) and 1% antibiotics (100 U/mL penicillin and 0.1 mg/mL streptomycin) at 37°C in a humidified atmosphere of 5% CO_2_. The detailed characteristics of AC16 were described by Mercy et al.^32^ On one hand, cells were plated in 6‐well plate and then transfected with 2 μg of constructed expression plasmids or empty vector pcDNA3.1 after 24 h using Lipofectamine™ 2000 transfection reagent (Invitrogen) according to the manufacturer's instructions. On the other hand, AC16 and HEK293T were plated in 24‐well plates at a density of 2.5 × 10^4^ cells per well and then co‐transfected with 0.8 μg of luciferase reporter plasmid, 50 ng of Renilla luciferase plasmid and 100 nmol of has‐miR‐133a‐3p (RIBOBIO Co., Ltd, Guangzhou, China) or mimics control. After 48 h of transfection, cells were harvested using the Passive Lysis Buffer (SIRIUS, Pforzheim, Germany). The levels of luciferase expression were adjusted with reference to Renilla luciferase activity and relative to the average values of wild‐type for corresponding variants. Each reporter was performed six independent experiments to avoid potential experimental errors.

### 2.6 Protein extraction and immunoblots

Cells were harvested 48 h after transfection and lysed with lysis solution (50 mmol/L Tris‐Cl, pH 8.0; 150 mmol/L NaCl; 0.02% sodium azide; 0.1% SDS; 1 μg/mL aprotinin; 1% Nonidet P‐40; and 0.5% sodium deoxycholate) containing protease inhibitors (100 μg/mL phenylmethylsulfonyl fluoride, 2 μg/mL aprotinin, 2 μg/mL leupeptin). Supernatant was collected after centrifuging at 12,000 × *g* for 20 min and protein concentrations were measured using the BCA protein assay reagent kit (Boster, China). A total of 20 μg of protein extracts for each samples were denatured in sample buffer (SDS polyacrylamide) containing β‐mercaptoethanol and electrophoretically resolved by 10% SDS‐polyacrylamide gels, followed by transferring to polyvinylidene difluoride membranes. Non‐specific binding sites were blocked with 5% non‐fat milk for 2 h at room temperature. Subsequently, the membranes were incubated with primary antibodies overnight at 4°C, followed by incubation with a peroxidase‐conjugated secondary antibody. Bands were visualized by enhanced chemiluminescence reagents (Pierce Chemical, Rockford, IL) and quantified by densitometry.

### 2.7 Transcription assays of the *PIK3CG* Gene and protein

#### 2.7.1 Levels in human heart samples

To assess the effect of functional variant on the *PIK3CG* expression, we collected 194 samples of peripheral blood lymphocytes from participants undergoing coronary angiography in total. Details about RNA isolation, mRNA transcription, and PCR conducted in this study have been described previously.^30^ Absolute quantification methods were used to measure the mRNA levels of PIK3CG and ACTB with each sample in triplicate. Relevant primer sequences and detailed characteristics of individuals are shown in Tables S4 and S5, respectively. Expression of *PIK3CG* relative to ACTB was compared between individuals with TT genotype and with CC or CT genotype.

In addition, a total of 17 human heart samples were used to examine PIK3CG protein expression. The study was approved by the Review Board of Tongji College of Medicine and conducted in accordance with the principles of the Helsinki Declaration. Written informed consents were obtained from all patients. Detailed clinical characteristics of patients are listed in Table S6.

#### 2.7.2 Determination of hsa‐miR‐133a‐3p expression levels

The expression of hsa‐miR‐133a‐3p was measured using quantitative RT‐PCR. Human normal tissues including heart, adrenal gland, small intestine, adipose, skin, muscle, lung and large intestine were obtained from distal normal tissue of tumour patients. Detailed procedures for RNA extraction and quantitative RT‐PCR have been described previously.^33^ The study was approved by the Review Board of Tongji College of Medicine and conducted in accordance with the principles of the Helsinki Declaration. Written informed consents were obtained from all patients.

#### 2.7.3 Analysis of cell apoptosis

The impact of PIK3CG on H/R‐induced apoptosis was assessed by terminal‐deoxynucleoitidyl transferase‐mediated nick end labeling (TUNEL) staining using a TUNEL fluorescence kit (Beyotime Biotechnology). Briefly, AC16 cells were firstly transfected with siRNAs (100 nm/L) or a PIK3CG‐overexpression plasmid in a six‐well plate and then re‐plated in a 24‐well plate 24 h later, followed by H/R treatment. For H/R induction, AC16 cells were cultivated in serum‐free DMEM in a hypoxia chamber with 5% CO_2_ and 95% N_2_ for 30 min, then reoxygenated in a humidified atmosphere with 5% CO_2_ and 95% air at 37°C for 1 h in DMEM supplemented with 10% FBS. Following 24 h of H/R treatment, all cells were subjected to TUNEL analysis following standard protocols. The stained apoptotic cells were visualized under the inverted fluorescence microscope (Leica Microsystems, Wetzlar, Germany).

## 3 MATERIALS

Anti‐PIK3CG and anti‐ACTB antibody were purchased from Cell Signalling Technology (#5405) and ABclonal Technology (AC026), respectively. The sequence of siRNAs targeting PIK3CG gene was as follows: CTGGCATTTTAGATACGAA, which was designed and synthesized by Guangzhou RiboBio Co., Ltd. TUNEL fluorescence kit was from Beyotime Biotechnology (C1088).

### 3.1 Statistical analysis

Statistical analyses were conducted with SPSS version 13.0 (SPSS, Inc, Chicago, Illinois) for Windows (Microsoft Corp, Redmond, Wash). Haploview version 4.1 was used for calculation of linkage disequilibrium. Data are expressed as means ± standard deviation (SD). Cox proportional hazards regression model was used for prognosis analysis of HF, with or without adjustment for traditional risk factors including age, sex, hypertension, diabetes, hyperlipidemia, smoking status and β‐blocker use. We performed all biostatistics calculations with Prism (GraphPad). Two independent samples were compared using Student's *t* test. In order to minimize the effect of extreme values, we used‐Logorithmic transformation of PIK3CG/ACTB for comparison of PIK3CG mRNA level between samples with different genotypes. All probability values were two‐sided and *p* < 0.05 was considered to be significant.

## 4 RESULTS

### 4.1 DNA re‐sequencing results, haplotype analysis of common variants within PIK3CG gene

**FIGURE 1:**
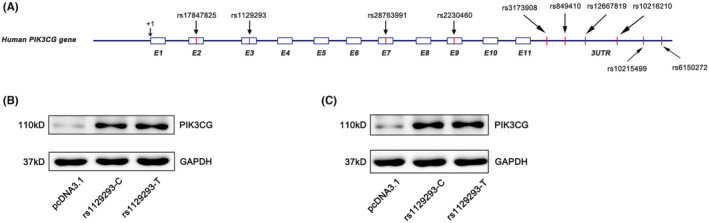
Functional analysis of rs1129293 synonymous variant. (A) Map of single‐nucleotide polymorphisms (SNPs) in the promoter and exon of *PIK3CG* genotyped in 48 healthy individuals. (B, C) AC16 and HEK293T cells were transfected with expression plasmids engineered to harbour either rs1129293‐C or –T allele. Empty vector pcDNA3.1 was served as control. The protein level of PIK3CG showed no difference between wild‐type and mutant‐type alleles.

By re‐sequencing 48 randomly selected unrelated healthy individuals from the Han Chinese population, we identified 12 common SNPs (minor allele frequency, MAF >0.05) in *PIK3CG* gene, including two in the promoter region, four in coding region, and six in 3′UTR (Table 2) (Figure 1A). All the variants were found to be in Hardy–Weinberg equilibrium in our population (*p* > 0.05). Considering a substantial of variants in *PIK3CG*, we attempt to narrow down the candidate SNPs with MAF >0.1. Subsequently, we conducted linkage analysis and identified three haplotype structures (Table 3).

**TABLE 2:** Characteristics of PIK3CG variants identified by Sanger sequencing.

dbSNPs	Gene position^a^	Gene region	MAF	Maj > min^b^
rs4730204	chr7:106505057	Promoter	0.344	T/C
rs4727666	chr7:106505478	Promoter	0.075	A/G
rs17847825	chr7:106509331	Missense	0.202	C/A
rs1129293	chr7:106513011	Synonymous	0.3	C/T
rs28763991	chr7:106522592	Missense	0.058	A/G
rs2230460	chr7:106524689	Synonymous	0.202	C/T
rs3173908	chr7:106546087	3UTR	0.303	C/T
rs849410	chr7:106546304	3UTR	0.058	T/G
rs12667819	chr7:106546657	3UTR	0.425	G/A
rs10216210	chr7:106547469	3UTR	0.303	G/C
rs10215499	chr7:106547921	3UTR	0.303	A/G
rs6150272	chr7:106548210	3UTR	0.303	−/AAC(AT)_4_GCTAA(TA)_4_

Abbreviation: MAF, minor allele frequency.

^a^ Base pair position is based on NCBI GRCH37.

^b^ With major allele given first, followed by minor allele.

**TABLE 3:** Haploblock structure of variants in *PIK3CG* gene with MAF >0.1 identified by Sanger sequencing.

Haploblock structure	Gene position^a^	dbSNP ID^b^	Gene region	Maj > Min^c^	MAF
Haploblock 1	chr7:106505057	rs4730204	Promoter	T/C	0.344
	chr7:106513011	rs1129293	Synonymous	C/T	0.3
	chr7:106546087	rs3173908	3UTR	C/T	0.303
	chr7:106547469	rs10216210	3UTR	G/C	0.303
	chr7:106547921	rs10215499	3UTR	A/G	0.303
	chr7:106548210	rs6150272	3UTR	‐/AAC(AT)_4_GCTAA(TA)_4_	0.303
Haploblock 2	chr7:106509331	rs17847825	Missense	C/A	0.202
	chr7:106524689	rs2230460	Synonymous	C/T	0.202
Haploblock 3	chr7:106546657	rs12667819	3UTR	G/A	0.425

Abbreviations: MAF, minor allele frequency; SNP, single nucleotide polymorphism.

^a^ Base pair position is based on NCBI GRCH37.

^b^ Polymorphism are numbered relative to transcription start site.

^c^ With major allele given first, followed by minor allele.

### 4.2 Association of tagged SNPs with the prognosis of HF

**FIGURE 2:**
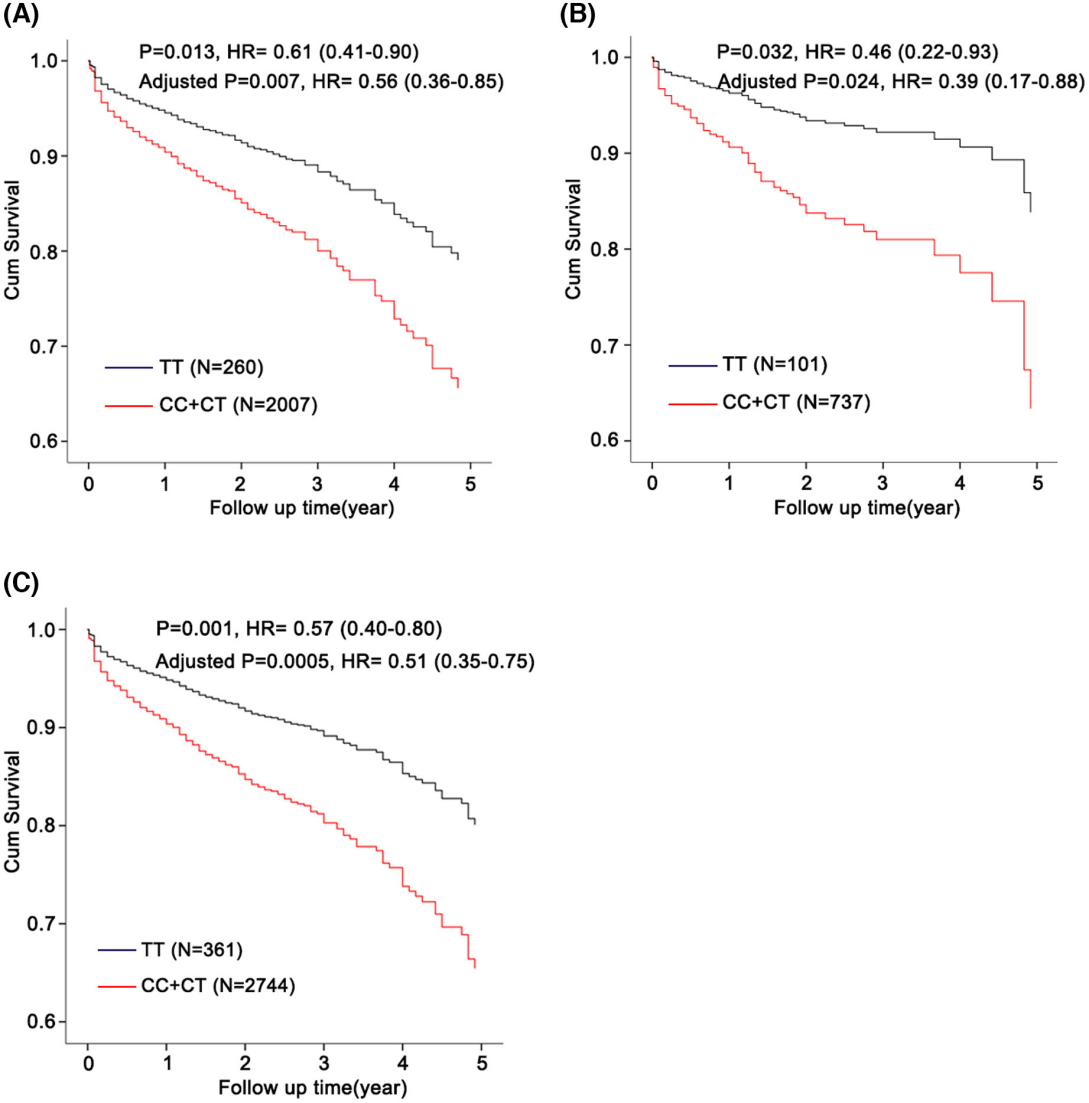
Effects of rs1129293 on the prognosis of HF patients. Cox proportional hazards models analysis showed that rs1129293 was associated with the prognosis of HF in the first cohort (A) replicated cohort (B) and combined cohort (C).

Firstly, we selected rs1129293, rs17847825 and rs12667819 as the tagged SNPs for genotyping from the corresponding haplotype. As shown in Table 4, only rs1129293 demonstrated a significant association with the prognosis of HF in recessive model within the first cohort (*p* = 0.013). The association remained statistically significant even after adjustment for traditional risk factors including sex, age, hypertension, diabetes, hyperlipidemia, smoking state, and β‐blocker use (*p* = 0.007, hazard ratio [HR] = 0.56) (Figure 2A). Subsequently, we attempt to confirm our discoveries in the replicated cohort. The results were consistent, with the rs1129293‐C allele being significantly associated with an increased risk of cardiovascular death and cardiac transplantation (adjusted *p* = 0.024, HR = 2.59) (Table 4, Figure 2B). Finally, we combined the two populations, and the results indicated that the rs1129293‐T allele was significantly associated with a more favourable prognosis compared to the C allele (Table 4, Figure 2C).

**TABLE 4:** Association of genetic polymorphisms in *PIK3CG* gene with HF prognosis.

	SNP	Genotype			MAF	Additive model			Dominant mode			Recessive mode		
						P	Adjusted *p*	HR (95% CI)	P	Adjusted *p*	HR (95% CI)	*p*	Adjusted *p*	HR (95% CI)
Discovery Population	rs1129293	CC (999)	CT (1008)	TT (260)	0.34	0.1	0.13	0.88 (0.75–1.04)	0.54	0.77	0.97 (0.78–1.20)	***0.013***	***0.007***	0.56 (0.36–0.85)
	rs17847825	CC (1475)	AC (711)	AA (81)	0.19	0.26	0.27	1.12 (0.92–1.36)	0.32	0.26	1.15 (0.91–1.45)	0.42	0.7	1.12 (0.64–1.95)
	rs12667819	GG (789)	AG (1098)	AA (380)	0.41	0.75	0.74	0.97 (0.81–1.16)	0.81	0.85	0.98 (0.75–1.27)	0.77	0.7	0.94 (0.67–1.31)
Replicated Population	rs1129293	CC (396)	CT (341)	TT (101)	0.32	0.13	0.24	1.18 (0.89–1.56)	0.54	0.94	1.02 (0.70–1.47)	***0.032***	***0.024***	0.39 (0.17–0.88)
Combined	rs1129293	CC (1395)	CT (1349)	TT (361)	0.33	0.03	0.06	0.87 (0.76–1.00)	0.41	0.76	0.97 (0.81–1.16)	***0.001***	***0.0005***	0.51 (0.35–0.75)

*Note*: Hazard ratio (HR) and 95% confidence intervals (95% CIs) were obtained using Cox regression, with and without adjustment for sex, age, hypertension, diabetes, hyperlipidemia, smoking status and β‐blocker use. Bold and italic represents the statistical significance.

Abbreviations: MAF, minor allele frequency; SNP, single‐nucleotide polymorphism.

### 4.3 SNP rs1129293 is associated with increase in *PIK3CG* expression

**FIGURE 3:**
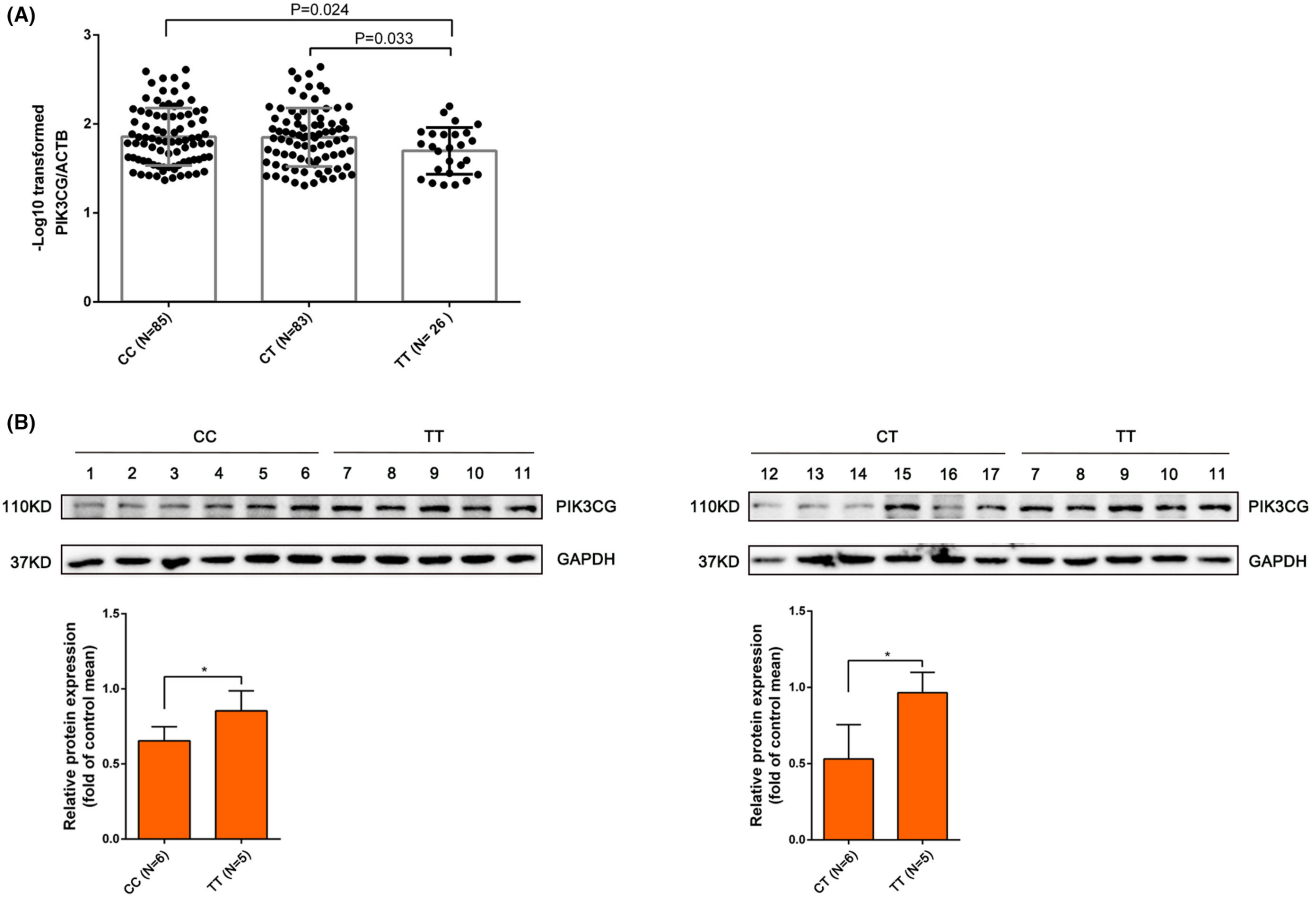
In vivo *PIK3CG* transcription and translation differs by genotype. (A) Absolute PIK3CG mRNA levels (relative to housekeeping gene *ACTB*) were measured in total RNA preparations from lymphocytes and compared among different genotypes. (B) Western blot analysis of 17 human failing heart samples showed the presence of rs1129293‐T allele increased protein expression of PIK3CG. *p < 0.05.

We then investigated the mechanism underlying the reduction in HF‐related mortality risk mediated by the variant rs1129293. Since rs1129293 is synonymous and 4 other SNPs in strong linkage disequilibrium with rs1129293 were all located in the 3′UTR, we attempted to explore whether the variant could affect *PIK3CG* expression. Firstly, we compared the *PIK3CG* mRNA relative expression in lymphocytes, including 194 samples (85 CC, 83 CT and 26 TT genotype). The result revealed that *PIK3CG* transcription was increased in the samples from individuals with the TT genotype compared with those with the CC and CT genotypes (Figure 3A), suggesting that the variant affects *PIK3CG* expression. Subsequently, we examined PIK3CG protein expression in 17 human heart tissues with different genotypes using western blotting. The results indicated that PIK3CG protein levels were higher in tissues with TT genotype compared with those with CC and CT genotypes (Figure 3B).

### 4.4 Association between rs1129293 and parameters of cardiac ultrasonography

Furthermore, we compared the cardiac ultrasound parameters among patients with different genotypes. As shown in Table 5, no significant differences were observed in terms of left ventricular end‐diastolic diameter (LVEDD), left atrium (LA), and interventricular septal thickness at diastole (IVSD) among patients with CC, CT, and TT genotype in the discovery population (*p* > 0.05). In the replicated population, individual with TT genotype exhibited a decrease in LVEDD compared to those with CC and CT genotypes (*p* < 0.05).

**TABLE 5:** Cardiac ultrasound parameters grouped by rs1129293.

Population	Genotype	LVEDD, mm	LA, mm	IVSD, mm
Discovery population	CC	56.7 ± 10.9	40.7 ± 7.9	10.2 ± 2.4
	CT	56.1 ± 11.0	41.1 ± 8.2	10.3 ± 2.8
	TT	56.3 ± 12.5	41.5 ± 8.6	10.2 ± 2.5
Replicated population	CC	57.7 ± 11.0	41.8 ± 7.8	10.2 ± 2.5
	CT	56.0 ± 11.2	40.5 ± 7.7	10.3 ± 2.8
	TT	52.9 ± 9.8	39.0 ± 7.7	9.9 ± 2.0

Abbreviations: IVSD, Interventricular Septal Thickness at Diastole; LA, left atrium; LVEDD, left ventricular end‐diastolic diameter.

### 4.5 Functional analysis of the cause SNP

**FIGURE 4:**
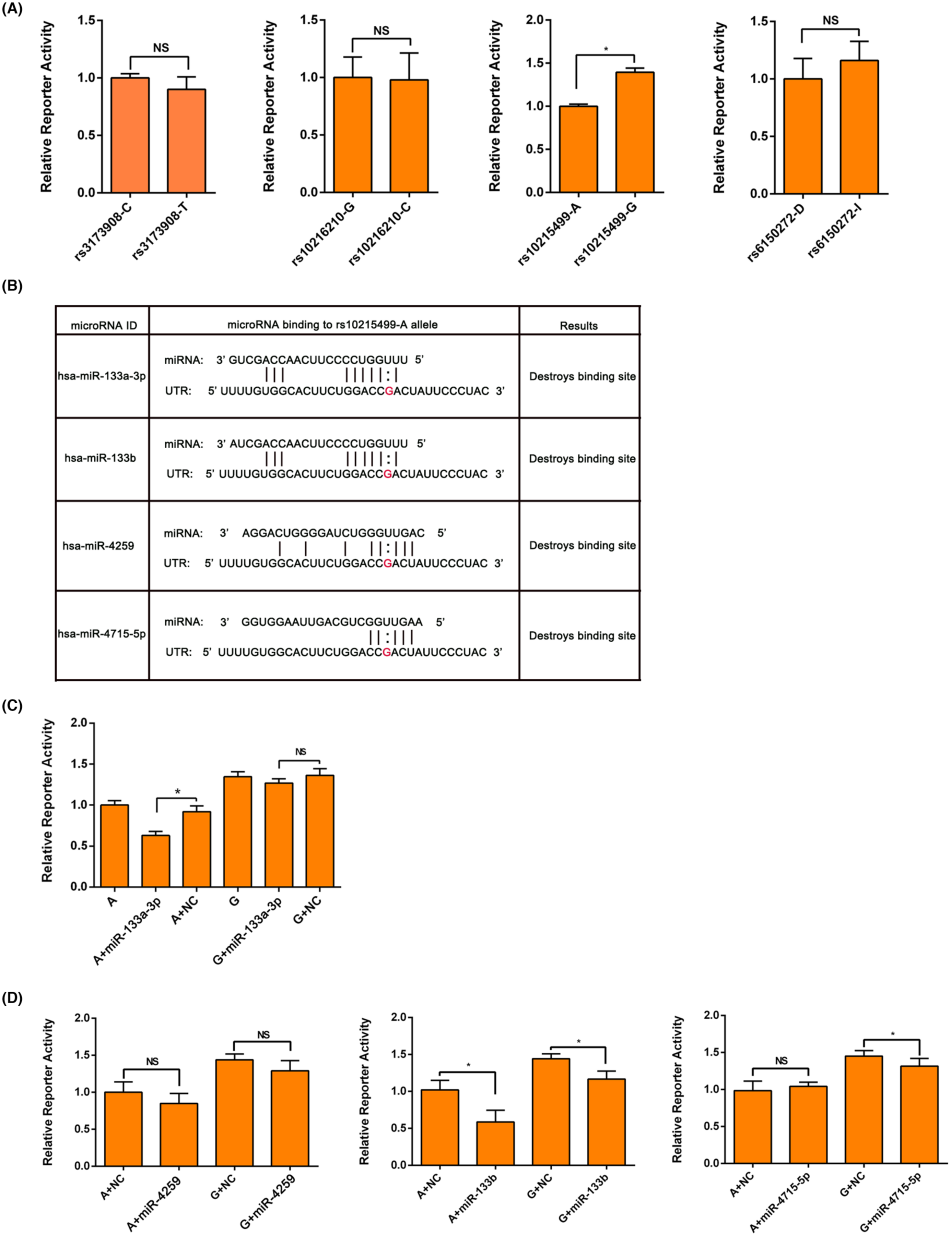
The G allele of rs10215499 destroys a binding site for microRNAs. (A) Luciferase assays showed that only rs10215499 displayed different transcriptional activity between wild‐type and mutant‐type alleles in AC16 cells. (B) Bioinformatic analyses showed that the allele G of rs10215499 destroyed a binding site for has‐miR‐133a‐3p, has‐miR‐133b, has‐miR‐4259 and has‐miR‐4715‐5p. (C) Luciferase assays showed that hsa‐miR‐133a‐3p displayed rs10215499‐A allele‐dependent inhibiting effect on the luciferase activity. While similar results were not observed for has‐miR‐4259, has‐miR‐133b and has‐miR‐4715‐5p (D). NS, not significant; *p < 0.05.

Considering the possibility that synonymous variants could also disturb the protein expression of corresponding gene,^34^ we attempt to explore the effect of rs1129293 on PIK3CG protein level. In AC16 and HEK293T cells, there was no difference in the expression level of PIK3CG between the rs1129293‐C and T alleles (Figure 1B,C). Subsequently, we performed luciferase assay to assess the remaining variants in the 3′UTR, which were in strict linkage disequilibrium with rs1129293. As shown in Figure 4A, the expression of the reporter gene with the rs10215499‐G allele significantly increased compared to the rs10215499‐A allele, and this finding was replicated in HEK293T cells (Figure S1). However, we did not observe any effect on luciferase activity in rs3173908, rs10216210 and rs6150272 luciferase assays in AC16 cells (Figure 4A).

These results suggest that rs10215499 may be the causal SNP responsible for the different prognosis of HF.

We used the online database miRNASNPv3 (https://guolab.wchscu.cn/miRNASNP//#!/) to determine the potential impact of rs10215499 on miRNA‐binding sites. The analysis revealed that four miRNAs, namely hsa‐miR‐133a‐3p, hsa‐miR‐133b, hsa‐miR‐4259, and hsa‐miR‐4715‐5p, were predicted to bind to the rs10215499‐A allele but not the G allele (Figure 4B). Subsequently, we performed luciferase activity assay and observed that only hsa‐miR‐133a‐3p exhibited rs10215499‐A allele‐dependent inhibitory effect on luciferase activity (Figure 4C). While other 3 miRNAs had no similar effects (Figure 4D).

**FIGURE 5:**
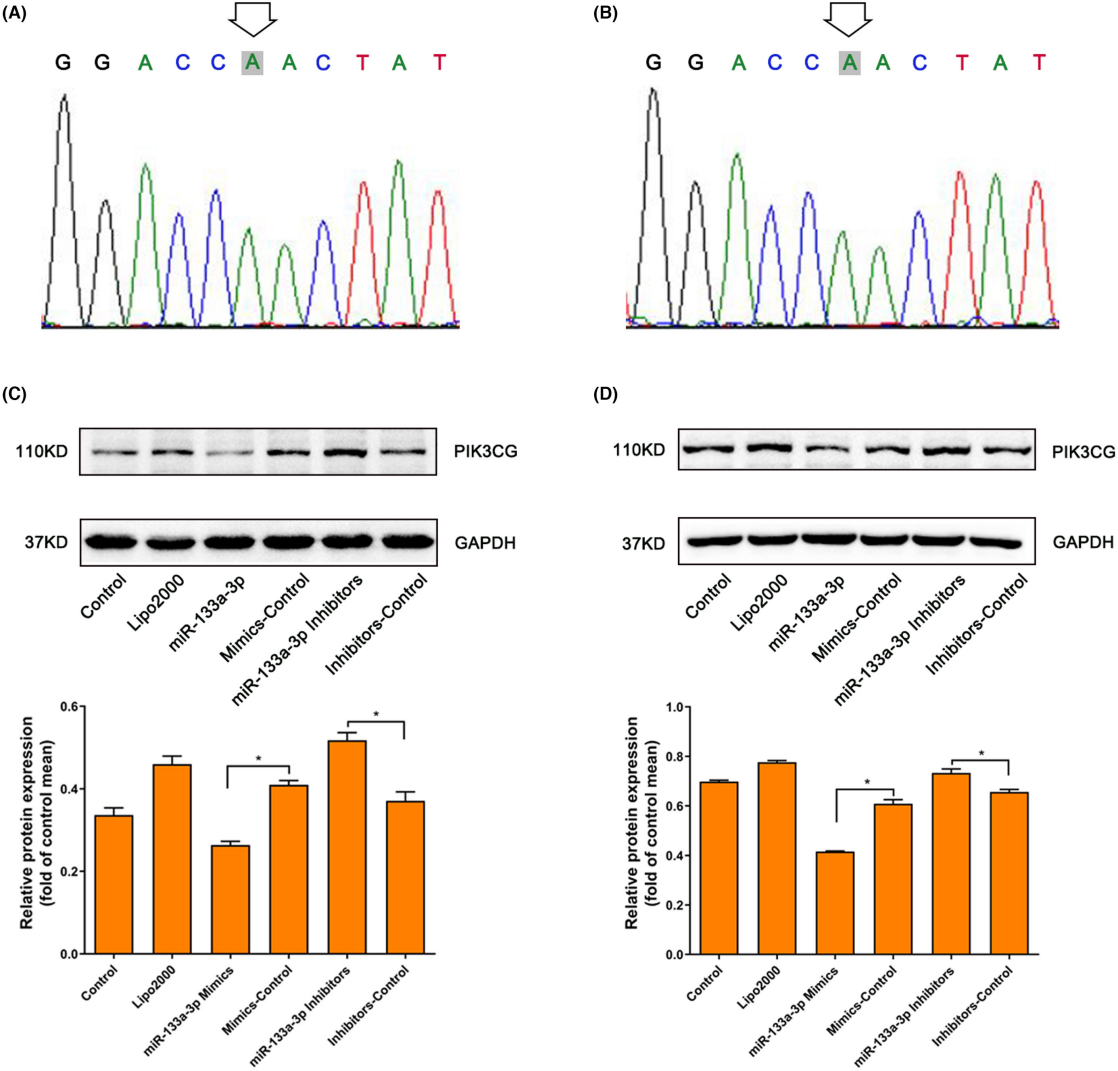
In vitro inhibitory effect of hsa‐miR‐133a‐3p on PIK3CG expression. (A, B) AC16 and HEK293T cells were identified to be rs10215499‐AA genotype by sanger sequencing. (C, D), hsa‐miR‐133a‐3p could significantly reduce PIK3CG protein levels in both AC16 and HEK293T cells, which could be reversed by miR‐133a‐3p inhibitors. *p < 0.05.

To further validate the effects of hsa‐miR‐133a‐3p on endogenous PIK3CG expression, we sequenced AC16 and HEK293T cell lines and found them to be rs10215499‐AA genotype (Figure 5A,B). Then we transfected the cells with mimics control, miR‐133a‐3p mimics, inhibitor control and miR‐133a‐3p inhibitor, respectively. Western blot results demonstrated that miR‐133a‐3p downregulated PIK3CG expression, and the inhibition of miR‐133a‐3p expression using its inhibitor significantly upregulated expression of PIK3CG in AC16 and HEK293T cells (Figure 5C,D). These findings collectively suggest that the rs10215499‐G allele may disrupt the binding site of hsa‐miR‐133a‐3p and consequently increase the expression of PIK3CG, ultimately contributing to improved cardio‐protection and prognosis for patients with heart failure.

### 4.6 Heart hsa‐miR‐133a‐3p level

**FIGURE 6:**
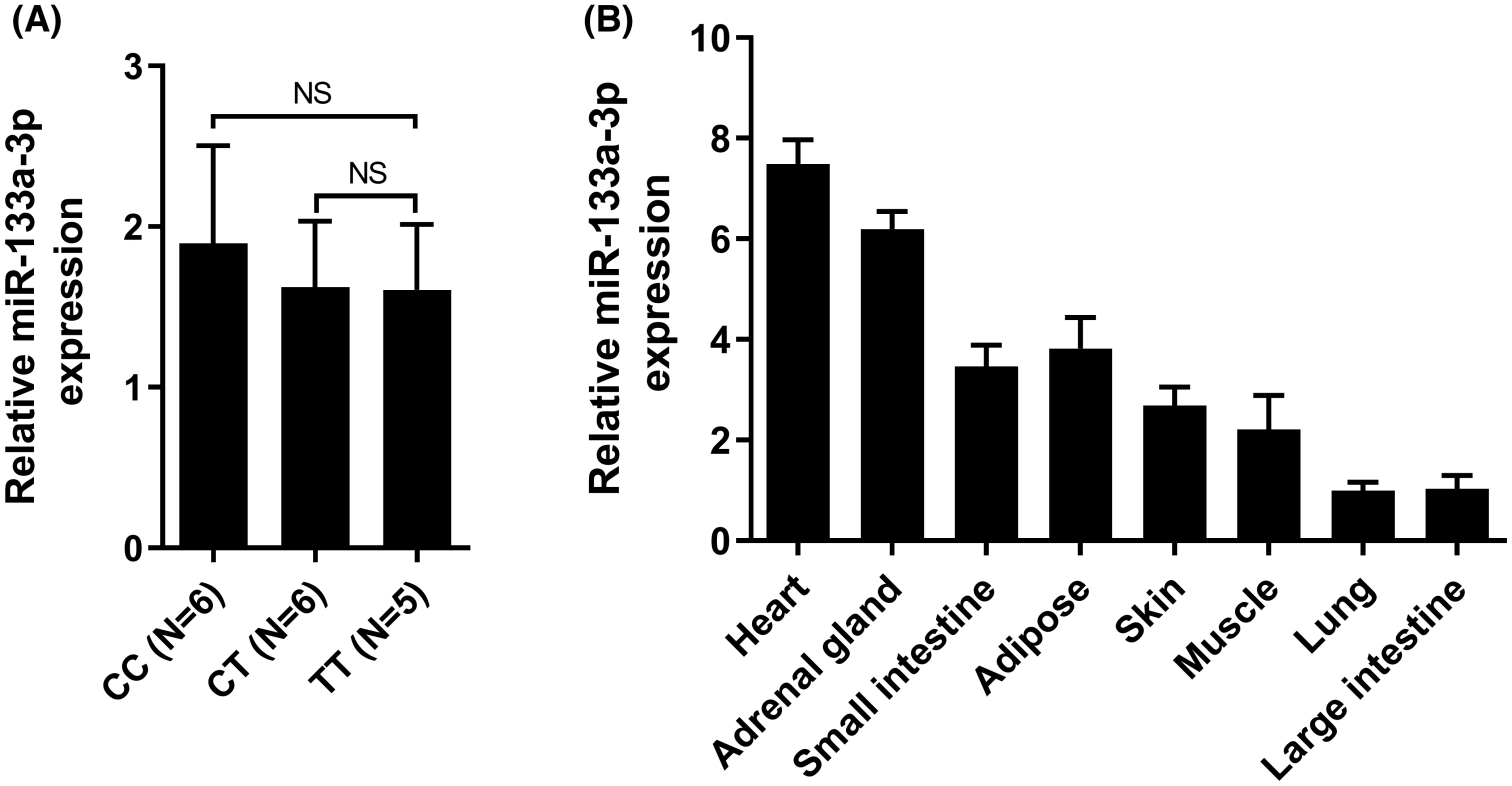
The expression level of hsa‐miR‐133a‐3p. (A) hsa‐miR‐133a‐3p level showed no difference in heart with different genotypes. (B) Comparison of hsa‐miR‐133a‐3p expression level in different human tissues. NS, not significant.

Real‐time quantitative PCR was performed for measurement of hsa‐miR‐133a‐3p. The results demonstrated that the expression level of hsa‐miR‐133a‐3p was consistent across heart tissues with different genotypes (Figure 6A). Additionally, hsa‐miR‐133a‐3p was found to be expressed in various human tissues, with the highest expression level observed in the heart (Figure 6B).

### 4.7 Apoptosis assay

**FIGURE 7:**
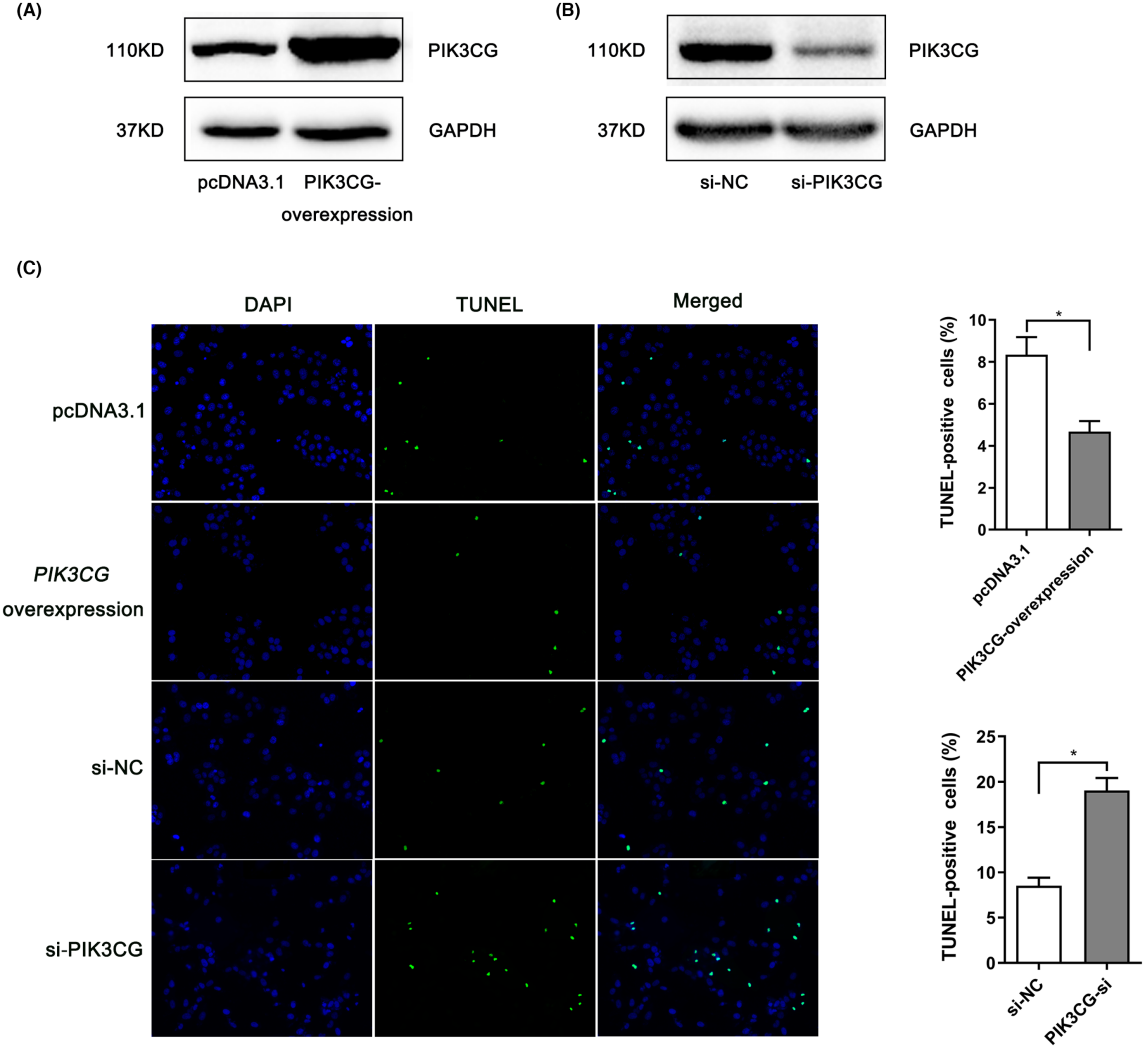
Effect of PIK3CG on H/R‐induced cardiomyocyte apoptosis in vitro. (A, B) PIK3CG‐overexpression plasmid and PIK3CG‐siRNAs showed significantly overexpression and inhibitory effects, respectively. (C) TUNEL assay detected TUNEL positive cells at 24 h after H/R treatment. *p < 0.05.

The biological function of PIK3CG under H/R conditions was investigated by overexpressing and silencing PIK3CG in AC16 cells. Firstly, the pcDNA3.1‐PIK3CG vector and PIK3CG‐siRNA could significantly increase and reduce the expression level of PIK3CG, respectively (Figure 7A,B). Following 24 h of H/R treatment, AC16 cells with PIK3CG overexpression exhibited a lower apoptosis rate compared to the control group (Figure 7C). Conversely, H/R‐induced apoptosis in AC16 cells was aggravated by PIK3CG‐siRNA transfection (Figure 7C). These findings demonstrated a protective role of PIK3CG in H/R‐induced apoptosis in cardiomyocytes in vitro.

## 5 DISCUSSION

Heart failure is a complex clinical syndrome with pathophysiological heterogeneity.^35^ There is substantial evidence suggesting the involvement of PI3Kγ in the pathological processes of heart failure.^9^, ^10^ In this study, we identified a common variant rs10215499 in the 3′UTR of *PIK3CG* gene associated with the prognosis of HF in both the first cohort (*p* = 0.013, HR = 0.56) and replicated cohort (*p* = 0.032, HR = 0.39). Importantly, this association remains significant even after adjusting for traditional risk factors, including age, gender, hypertension, diabetes, hyperlipidemia, smoking status, and the use of β‐blockers.

As a catalytic subunit, PI3Kγ formed a heterodimer with p87 or p101 subunit and has been extensively studied in various physiological systems, including immune, cardiovascular, endocrine, neuronal functions, as well as its role in malignancy.^36^ Importantly, the function of PI3Kγ in the myocardium has gained considerable attention due to its abundant expression in cardiovascular tissue.^9^ Numerous studies have demonstrated the protective effects of PI3Kγ against myocardial injury and remodelling induced by I/R and TAC, with these effects being abolished in PI3Kγ knockout mice.^9^, ^10^, ^22^ Our in vitro investigation also demonstrated that PI3Kγ confers resistance to H/R‐induced apoptosis in AC16 cells, consistent with the findings of Mauro et al., who reported that PI3Kγ knockout significantly increased cardiomyocyte apoptosis in a mouse model of myocardial infarction (MI).^9^ Furthermore, increased expression levels and activity of PI3Kγ were observed in the myocardium of mice with TAC‐induced heart failure and in patients with heart failure.^18^, ^22^ Considering the important role of PI3Kγ in myocardium, we attempted to investigate the association of variants in *PIK3CG* gene with the prognosis of HF. Interestingly, we identified a common SNP located in the 3′UTR that displayed strong association with the prognosis of HF in both first (adjusted *p* = 0.007, HR = 1.80) and replicated (adjusted *p* = 0.024, HR = 2.59) cohorts. Importantly, this association was independent of current known risk factors as the statistical significance remained after adjustment for sex, age, hypertension, diabetes, hyperlipidemia, smoking state, and β‐blocker use.

To date, numerous genetic investigations have been conducted on the *PIK3CG* gene. For instance, rs4288294 and rs116697954 in *PIK3CG* have been reported to be associated with plasma HDL‐cholesterol concentrations.^15^ In a case–control study, Gu et al. identified an association between rs12667819 in 3′UTR of *PIK3CG* and Attention‐deficit/hyperactivity disorder (ADHD).^37^ Additionally, a positive correlation between *PIK3CG* SNPs (rs1129293 and rs17398575) and patients with poor responsiveness to clopidogrel may exist.^29^ However, no investigation has been conducted to explore the role of genetic variants in *PIK3CG* in HF. Our study was the first to demonstrate the association of variant in *PIK3CG* with the prognosis of HF. Furthermore, detailed functional assays were conducted to illustrate the underlying mechanism, which were lacking in the previous genetic studies on *PIK3CG*.

As a lipid and protein kinase, PI3Kγ has been extensively studied in both cardiac cells and leukocytes.^38^ The role of kinase functions and kinase‐independent functions of PI3Kγ in myocardium showed completely opposite effects.^23^ Numerous studies have demonstrated the protective role of kinase‐independent scaffolding function of PI3Kγ in heart failure.^10^, ^19^, ^21^ In contrast, its kinase function appears to be detrimental,^21^, ^25^ leading to controversies in the field.^9^, ^23^ Overall, PI3Kγ confers cardio‐protection in response to various pathological stimuli. Importantly, our study revealed that individuals with homozygous mutation had higher *PIK3CG* expression in both protein levels of human heart and mRNA levels of peripheral blood lymphocytes. These individuals with homozygous mutation also exhibited a favourable prognosis for HF, aligning with the beneficial role of PI3Kγ observed in mice.^10^

**FIGURE 8:**
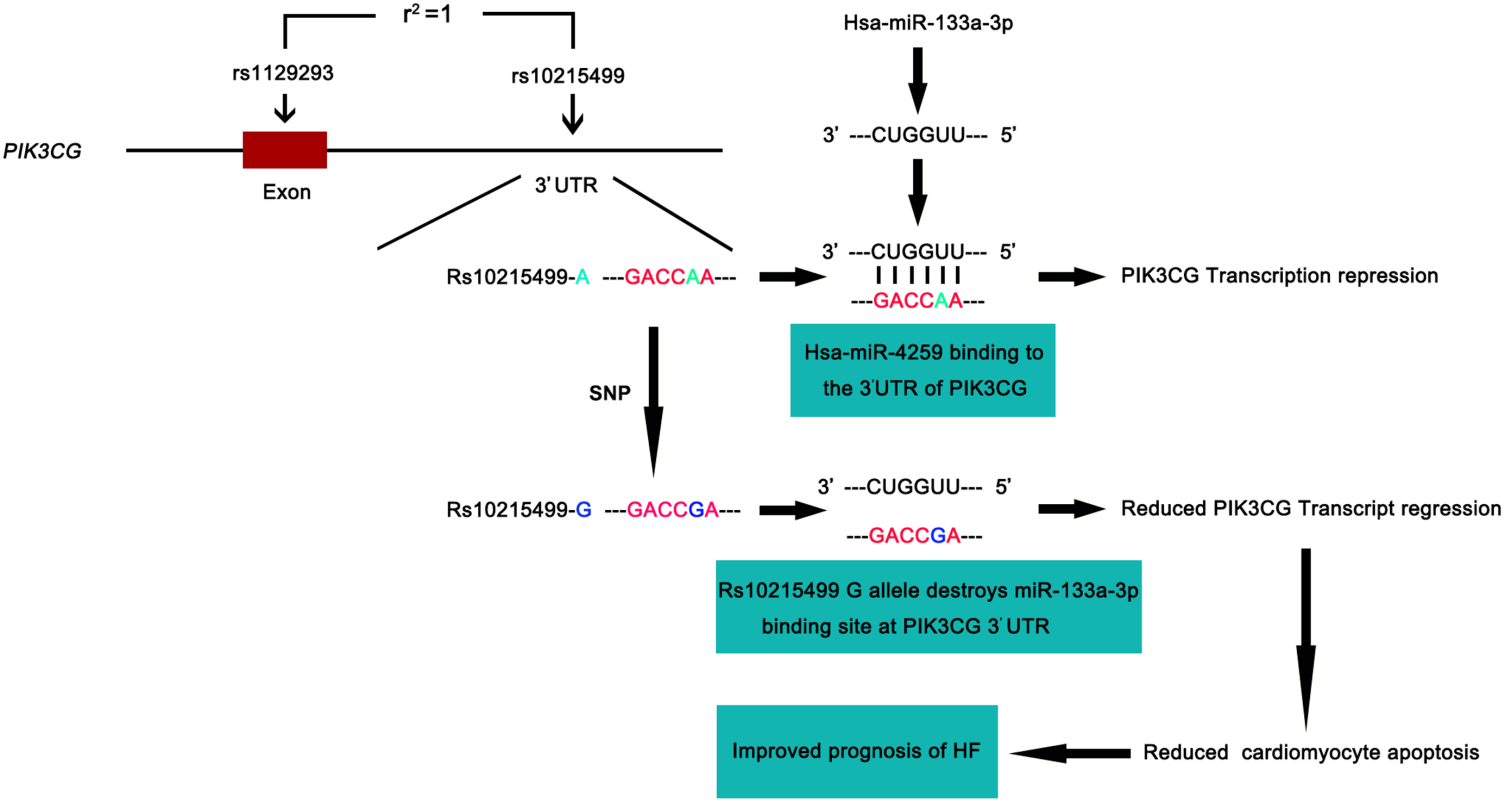
Role of hsa‐miR‐133a‐3p in the Regulation of *PIK3CG* Transcription. The variant rs10215499, which is in strict LD with genotyped synonymous SNP rs1129293, is a A‐ to ‐G change in the 3′UTR of *PIK3CG* and located in the predicted binding site of hsa‐miR‐133a‐3p. Rs10215499‐G allele destroys the hsa‐miR‐133a‐3p binding site and subsequently results in increased transcription and translation of PIK3CG, followed by reduced cardiomyocyte apoptosis under H/R condition, which ultimately favours better prognosis of HF.

Furthermore, our study revealed that rs10215499‐G allele destroyed the binding site of hsa‐miR‐133a‐3p, resulting in increased transcription of PIK3CG. Consequently, this transcriptional upregulation contributes to a more favourable prognosis for heart failure (Figure 8). Additionally, we observed no significant difference in the level of hsa‐miR‐133a‐3p among heart tissues with different genotypes, with the heart exhibiting the highest level of miR‐133a‐3p. Our study indicated that hsa‐miR‐133a‐3p could reduce the expression of PIK3CG and suggested a potential involvement of hsa‐miR‐133a‐3p in cardiac pathophysiology, which needs further investigation in the future.

There are still some limitations in our study. First, we only focused on functional variants in *PIK3CG* gene. Other SNPs in linkage disequilibrium with rs10215499 may also affect the prognosis of HF and need further investigation. Second, there may be additional regulatory factors involved in the regulation of PIK3CG gene expression besides hsa‐miR‐133a‐3p. Finally, the MAF of rs10215499 varies significantly among different racial groups, being highest in the Asian population and lowest in the European population. It is important to note that our study results may not be generalizable to other racial groups.

In conclusion, we have identified a common variant in *PIK3CG* gene significantly associated with the prognosis of HF in our discovery population, which was further validated in the replicated population. A series of functional assays indicated that rs10215499‐G allele may destroy the binding site of hsa‐miR‐133a‐3p, leading to increased transcription of PIK3CG and ultimately resulting in a better prognosis (Figure 8). Additionally, individuals with rs10215499‐GG genotype exhibited higher mRNA and protein level of PIK3CG compared to those with AA or AG genotype, which supported the aforementioned functional investigations. Furthermore, overexpression of PIK3CG could protect AC16 from H/R‐induced apoptosis, whereas silencing of PIK3CG had the opposite effect. Moreover, the high hazard ratios (HR = 1.96, 95% CI = 1.34–2.85; *p* = 0.0005) and high frequency of rs10215499 (MAF = 0.34) suggest that this SNP may account for a substantial proportion of poor prognosis of HF patients. Novel strategy through targeting PIK3CG is a promising way to decrease HF‐associated mortality and improve the prognosis of HF.

## AUTHOR CONTRIBUTIONS

**Dong Hu:** Conceptualization (lead); data curation (lead); formal analysis (lead); funding acquisition (lead); software (lead); validation (lead); writing – original draft (lead). **Lei Xiao:** Conceptualization (supporting); investigation (equal); methodology (lead); project administration (equal); resources (equal); software (equal); supervision (equal); validation (equal); visualization (equal). **Shiyang Li:** Writing – review and editing (equal).

## FUNDING INFORMATION

This work was supported by the project from Department of Science and Technology of Hubei Province (2023AFB464), Wuhan Science and Technology Bureau (2023020201020538), and The Central Hospital of Wuhan (22YJ21).

## CONFLICT OF INTEREST STATEMENT

The authors declared no competing interests for this work.

## Supporting information


**Figure S1.** Luciferase assays showed that rs10215499 displayed different transcriptional activity between wild‐type and mutant‐type alleles in HEK293T cells.

**Table S1.** Sequences of PCR primers used for amplification and sequencing of PIK3CG.

**Table S2.** Sequence of probes and primers sets.

**Table S3.** Sequence of primers for plasmids construction.

**Table S4.** Sequences of primers used for quantitative RT‐PCR.

**Table S5.** Baseline characteristics of 194 individuals undergoing coronary angiography.

**Table S6.** Clinical characteristics of HF patients.

## Data Availability

The datasets used and/or analysed during the current study are available from the corresponding author on reasonable request.
