# Supplementary figures and images for: A common variant in PIK3CG gene associated with the prognosis of heart failure
Source: J Cell Mol Med. 2024 Sep 8;28(17):e70069. doi: 10.1111/jcmm.70069 (PMC11381188; doi:10.1111/jcmm.70069)

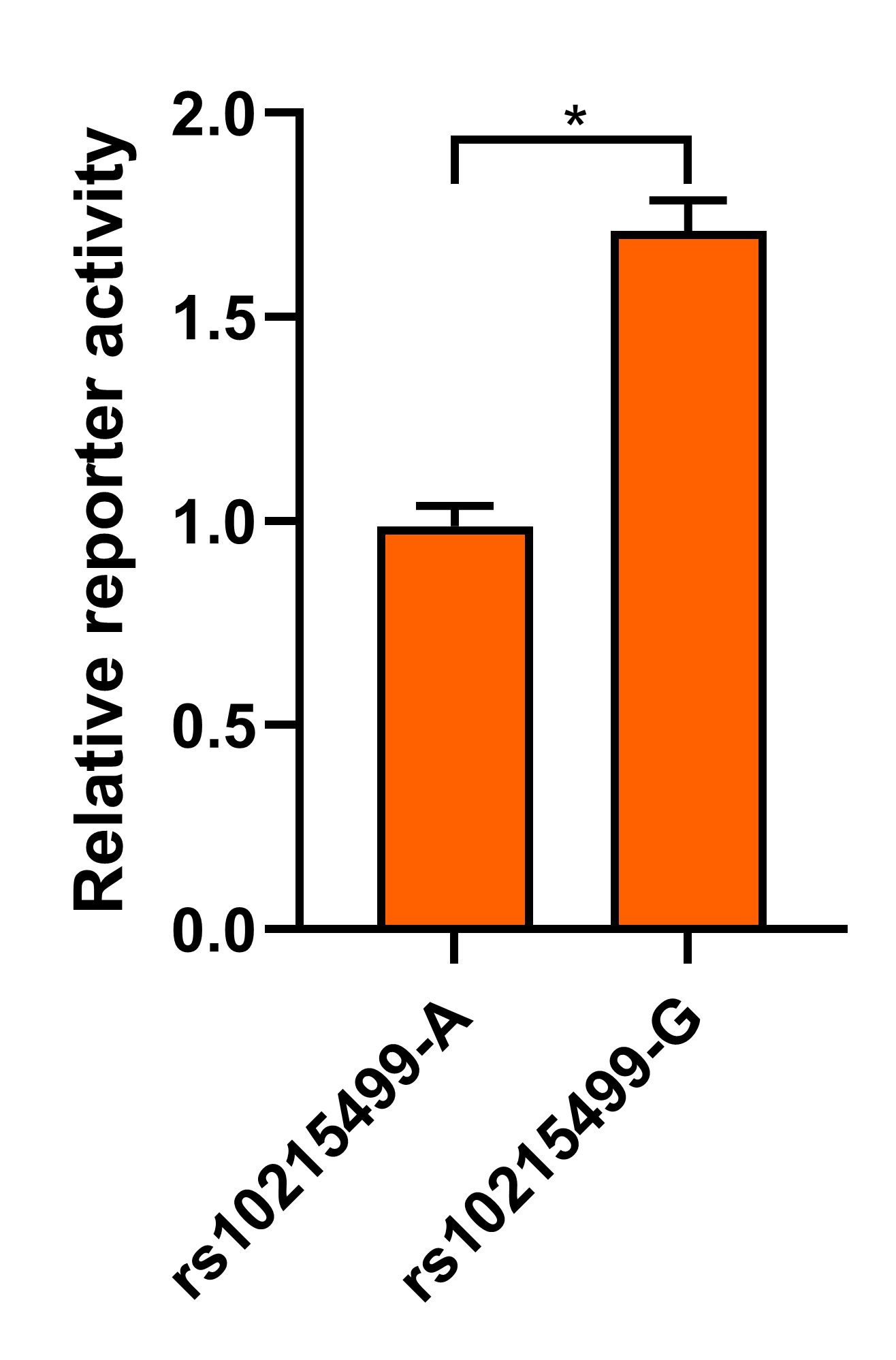

Supplement: Supplementary file 1 — Figure S1. Luciferase assays showed that rs10215499 displayed different transcriptional activity between wild‐type and mutant‐type alleles in HEK293T cells. [file JCMM-28-e70069-s002.tif]
